# Characterization and genomic study of EJP2, a novel jumbo phage targeting antimicrobial resistant *Escherichia coli*

**DOI:** 10.3389/fmicb.2023.1194435

**Published:** 2023-05-12

**Authors:** Dohyeong Jo, Hyeongsoon Kim, Yoona Lee, Jinshil Kim, Sangryeol Ryu

**Affiliations:** Department of Agricultural Biotechnology and Department of Food and Animal Biotechnology, Research Institute of Agriculture and Life Sciences, Seoul National University, Seoul, Republic of Korea

**Keywords:** bacteriophage, jumbo phage, antimicrobial resistance, *Escherichia coli*, antimicrobial agent, genome, phylogenetic analysis

## Abstract

The emergence of antimicrobial resistance (AMR) *Escherichia coli* has noticeably increased in recent years worldwide and causes serious public health concerns. As alternatives to antibiotics, bacteriophages are regarded as promising antimicrobial agents. In this study, we isolated and characterized a novel jumbo phage EJP2 that specifically targets AMR *E. coli* strains. EJP2 belonged to the *Myoviridae* family with an icosahedral head (120.9 ± 2.9 nm) and a non-contractile tail (111.1 ± 0.6 nm), and contained 349,185 bp double-stranded DNA genome with 540 putative ORFs, suggesting that EJP2 could be classified as jumbo phage. The functions of genes identified in EJP2 genome were mainly related to nucleotide metabolism, DNA replication, and recombination. Comparative genomic analysis revealed that EJP2 was categorized in the group of Rak2-related virus and presented low sequence similarity at the nucleotide and amino acid level compared to other *E. coli* jumbo phages. EJP2 had a broad host spectrum against AMR *E. coli* as well as pathogenic *E. coli* and recognized LPS as a receptor for infection. Moreover, EJP2 treatment could remove over 80% of AMR *E. coli* biofilms on 96-well polystyrene, and exhibit synergistic antimicrobial activity with cefotaxime against AMR *E. coli*. These results suggest that jumbo phage EJP2 could be used as a potential biocontrol agent to combat the AMR issue in food processing and clinical environments.

## Introduction

*Escherichia coli* is a main opportunistic pathogen that commonly colonizes in the gastrointestinal tract of both animals and humans (Kim et al., 2021), causing a range of intestinal and extra-intestinal disease. Various antibiotics have been applied orally or via injection to control this bacteria, but *E. coli* species have represented a capability to easily acquire the resistance genes by horizontal gene transfer (Poirel et al., 2018). Antimicrobial resistance (AMR) *E. coli*, such as Extended-Spectrum Beta-Lactamase (ESBL)-producing *E. coli*, exhibit a wide spectrum of resistance against all *β*-lactam antibiotics and other class of antibiotics such as fluoroquinolones, aminoglycosides (Coque et al., 2008; Dd Pitout, 2013). The emergence and prevalence of AMR in *E. coli* has become serious public health threats (Roca et al., 2015) and a number of studies have reported the isolation of AMR *E. coli* from patients, animals, and food chain (Rupp and Fey, 2003; Poirel et al., 2018; Park et al., 2019; Martak et al., 2022). Hence, World Health Organization (WHO) declared that AMR *E. coli* is one of the most urgent strains for which novel antimicrobials are needed (Shrivastava et al., 2018). Currently, the use of antibiotics is limited due to rapid acquisition of AMR in *E. coli* (Zurfuh et al., 2016; Parvez and Khan, 2018; Wu et al., 2018), so the development of alternative antimicrobial agents is necessary to address these concerns.

Bacteriophages (phages) are viruses that infect specific bacterial species and are the most abundant biological entities on earth (Bragg et al., 2014). In comparison to conventional antibiotics, phages have several advantages for use as alternative antimicrobial agents, such as harmlessness to human and commensal bacteria, host specificity, and low cost for production compared to development of novel antibiotics (Loc-Carrillo and Abedon, 2011). Phages are classified into two categories based on their life cycles; virulent and temperate phage (Kutter and Sulakvelidze, 2004) and the use of virulent phage is considered as a suitable strategy to control pathogens (Hassan et al., 2021). After the phage products, Listshield (Intralytix, Inc., Baltimore, MD, United States), was firstly approved by U.S. Food and Drug Administration (FDA) in 2006, a variety of phage products have commodified to prevent *E. coli* infection (Huang et al., 2022). *E. coli* phages (coliphages) are commonly isolated from environment. Although many studies of coliphages in terms of genetics and molecular biology have provided insights into the phage biology (Kutter and Sulakvelidze, 2004), but what we know about phage is just the tip of the iceberg.

Phages with a genome size from 200 to 500 kb are classified as jumbo phages (Yuan and Gao, 2017). There are over 22,000 registered phages in the NCBI database, and out of these, 581 phages can be classified as jumbo phages. Jumbo phages have distinct characteristics compared to phage with genome size under 200 kb. Nazir et al. (2021) reported that most jumbo phages morphologically represent head and tail sizes of over 100 nm (Nazir et al., 2021). Jumbo phages possess genomes that contain numerous genes associated with genome replication, modification and nucleotide metabolism, enabling them to replicate independently from the host. Genes with similar function in jumbo phage genomes are typically dispersed or organized into sub-clusters throughout the genome (Ceyssens et al., 2014; Guan and Bondy-Denomy, 2020). Transcription of early phage genes is commonly regulated by phage-encoded RNA polymerases (RNAPs) (Mesyanzhinov et al., 2002; Leskinen et al., 2016; Imam et al., 2019), and non-virion RANP is responsible for transcription of middle or late phage genes (Orekhova et al., 2019). In addition, jumbo phages possess a range of tRNAs and aminoacyl-tRNA synthetases, which can replace cleaved host tRNA to maintain translation of viral proteins. Iyer et al. (2021) analyzed that the number of tRNA genes in jumbo phages ranges from 4.5 to 22 per genome. The abundance of transcription and translation-related genes enables the post-infection development of jumbo phages, which implies a high level of independence from the host molecular machinery (Ceyssens et al., 2014; Lavysh et al., 2016). This independence appears to confer broad host ranges to jumbo phages, as in the case of *Xanthomonas citri* jumbo phage XacN1 (Yoshikawa et al., 2018).

In this study, we isolated and characterized *E. coli* jumbo phage EJP2, which has a genome size of 349,185 bp (accession no. OQ411014) with low sequence homology to the other jumbo phages currently known. We investigated its biological and genomic features of EJP2, and revealed that EJP2 recognized LPS as a phage receptor. EJP2 presented a broad host range, biofilm removal activity and exhibited synergistic antimicrobial efficacy against AMR *E. coli* when used in combination with cefotaxime (CTX) treatment. These results would help to expand our knowledge of jumbo phages and suggest the potential of EJP2 as alternative antimicrobial agents for biocontrol of AMR *E. coli*.

## Materials and methods

### Bacterial strains, plasmids and growth conditions

The bacterial strains used in this study are listed in Table 1. All bacteria were grown in Luria-Bertani (LB) broth and agar plates at 30°C and/or 37°C supplemented with appropriate antibiotics: ampicillin (Amp), 50 μg/ml; carbenicillin (Car), 100 μg/ml; kanamycin (Kan), 50 μg/ml; acridine orange (AO), 100 μg/ml, while isopropyl β-d-1-thiogalactopyranoside (IPTG) was added at a concentration of 50 or 100 μM.

**Table 1:** Bacterial strains and plasmids used in this study.

Strain and plasmid	Genotype and main characteristics^a^	Reference
*Escherichia coli*		
DH5α λ*pir*	ΦΔM15 Δ(*lacZYA-argF*) U169 *recA1 hsdR17 thi-1 supE44 gyrA96 relA1*/λ*pir*	Platt et al. (2000)
FORC82	Host for phages	This study
PS01	*β*-lactam antibiotics sensitive mutant of FORC82	This study
PS01 + pUHE	PS01 *+* pUHE21-2 *lacI*^q^	This study
PRS07	PS01 *waaR*::Tn5	This study
PRS07 + pUHE	PRS07 *+* pUHE21-2 *lacI*^q^	This study
PRS07 + pFORC82_*waaR*	PRS07 *+* pUHE21-2 *lacI*^q^::*waaR*	This study
AMR *E. coli* isolate 62		Kim et al. (2021)
Plasmids		
pUHE21-2*lacI*^q^	rep_pMB1_ *lacI*^q^; inducible Lac promoter; AmpR	Soncini et al. (1995)
pFORC82_*waaR*	pUHE21-2 *lacI*^q::^::*waaR*	This study
pKD13	*oriRγR6k bla FRT::kan::FRT*;KanR	Datsenko and Wanner (2000)

^a^Amp^R^, ampicillin resistant; Kan^R^, kanamycin resistant.

### Bacteriophage isolation and propagation

Animal fecal samples were collected in Seoul, South Korea. Phage isolation was conducted as previously described with some modifications (Saad et al., 2019). Sample was homogenized with 50 ml of sodium chloride-magnesium sulfate (SM) buffer (50 mM Tris–HCl, pH 7.5, 100 mM NaCl and 8 mM MgSO_4_·7H_2_O). Large particles were excluded by centrifuge (5,000 × *g*, 10 min, 4°C) and chloroform was added to supernatant for removal of residual bacteria. After centrifugation, supernatants were discarded to remove small phages and the pellet was suspended with 5 ml of SM buffer. This process was repeated three times. The resultant lysate was spotted on the bacterial lawn containing host cell. Briefly, 100 μl of cultured host cell (*E. coli* FORC82) was inoculated into 5 ml of LB soft agar [0.3% (supplemented with appropriate antibiotic and IPTG, if necessary)]. Mixture was poured on LB agar plates and solidified for 30 min. Ten microliters of serially diluted (10-fold) phage lysates were spotted on the bacterial lawn and dried for 20 min at room temperature. The plates were incubated at 30°C for at least 12 h to obtain single plaques. Small single plaques were picked with the sterile tip and eluted in 250 μl of SM buffer for further purification. This purification step was repeated at least three times. For phage propagation, host strain was incubated at 30°C for 2 h 30 min. The phage lysate was added into bacterial culture at a multiplicity of infection (MOI) of 1 and incubated for 4 h. The propagated phages were precipitated with polyethylene glycol (PEG) 6,000 and concentrated by CsCl density gradient ultracentrifugation (78,500 × *g*, 2 h, 4°C) (Kim H. et al., 2019).

### Morphological analysis by TEM

Each purified phage stock dilutions (4 μl, approximately 10^9^ PFU/mL) were placed on carbon-coated copper grids for 60 s and the excess phage was removed with filter paper. Equal volume of 2% aqueous uranyl acetate (pH 4.0) were added for 90 s to negatively stain the phage particles. Phages were examined by transmission electron microscopy (TEM; LEO 912AB transmission electron microscope; Carl Zeiss, Wezlar, Germany) at a 120-kV accelerating voltage, and images were scanned at the National Instrumentation Center for Environmental Management (Seoul, South Korea). Phage were morphologically classified according to the guidelines of the International Committee on Taxonomy of Viruses (ICTV) (Walker et al., 2022).

### Bacteriophage spot assay

The bacterial lawn was prepared as described by (Kim and Ryu, 2011). Briefly, 200 μl of cultured host cells was inoculated into 5 ml of LB soft agar [0.3% agar (supplemented with the appropriate antibiotic and IPTG, if necessary)]. This mixture was poured onto LB agar plates and solidified for 30 min. Ten microliters of serially diluted (10-fold) phage lysates were spotted on the bacterial lawn and dried at room temperature for 20 min. The plates were incubated for 12 h at 30°C, and the phage plaques were monitored.

### Bacterial challenge assays

One milliliter of LB broth was inoculated with overnight culture of *E. coli* FORC82. Bacterial cultures were incubated at 30 and 37°C for 2 h and infected with phage EJP2 at a MOI of 1. Optical density at 600 nm was measured every 1 h after phage infection until 12 h. SM buffer was added as a negative control. The experiments were conducted in triplicate.

### Sequencing of phage DNA and bioinformatics analysis

Phage genomic DNA was extracted by phenol-chloroform method as previously described (Sambrook and Russell, 2001). The purified phage DNA was sequenced using the Illumina Miseq platform (Illumina, San Diego, CA, United States) and assembled with the SPAdeS v.3.13.0 (Bankevich et al., 2012) at Sanigen Inc., South Korea. The ORFs were predicted by using Glimmer3 (Delcher et al., 2007), GeneMarkS (Besemer et al., 2001), and RAST annotation server^1^ (Aziz et al., 2008; Overbeek et al., 2014). The annotated data were assorted and arranged by using Artemis (Carver et al., 2008). The tRNA sequence in the phage genome were analyzed by tRNAscan-SE program (Lowe and Eddy, 1997). The functions of phage proteins were predicted by using NCBI BLASTp and InterProscan program (Altschul et al., 1990; Jones et al., 2014). Sequence alignment among EJP2 and other phages was performed using ClustalW (Thompson et al., 1994) using amino acid sequences of phage terminase large subunit (TerL), major capsid protein (MCP) and portal vertex protein. Relationships among the phage genome sequences were inferred using neighbor-joining method (Saitou and Nei, 1987) and the phylogenetic tree was constructed using MEGA-X v10.0.5 (Kumar et al., 2018). The bootstrap value of 5,000 replicates represented the evolutionary history of the analyzed taxa (Felsenstein, 1985). The evolutionary distances were represented using p-distance method (Nei and Kumar, 2000). Dot plot analysis was conducted using Gepard v1.40 (Krumsiek et al., 2007).

**Table 2:** Oligonucleotides used in this study.

Oligonucleotide	Sequence (5′ to 3′)	Purpose
Tn_pKD13_F	CTG TCT CTT ATA CAC ATC TTG TAG GCT GGA GCT TCG	Gene (*kanR*) amplification
Tn_pKD13_R	CTG TCT CTT ATA CAC ATC TCT GTC AAA CAT GAG AAT TAA TTC C	Gene (*kanR*) amplification
PRS07_confirm_F	TGG CAT GAA GCA AAT TTG ACA C	Sequence confirmation
PRS07_confirm_R	GAA GTT ATG CCT TTT ATA TAC TCA C	Sequence confirmation
pUHE21-2_F	GGA TCC TCT CAT AGT TAA TTT CT	Plasmid construction
pUHE21-2_R	AAG CTT AAT TAG CTG AGC TTG G	Plasmid construction
*waaR*_comple_F	AGA AAT TAA CTA TGA GAG GAT CCA TGA ATG AAT TTA TAA AAG AAC GGT TTT	Plasmid construction
*waaR*_comple_R	CCA AGC TCA GCT AAT TAA GCT TTT ATT TCT TAA GCT TGT ACT TAA TTA ATG	Plasmid construction

### Acridine orange and PNA treatment for curing the plasmids

Acridine orange (AO) was treated to *E. coli* FORC82 in order to cure the plasmids of *E. coli* FORC82 (Hirota, 1960). An overnight culture of *E. coli* FORC82 was sub-cultured to 3 ml of fresh LB broth with AO (100 μg/ml) and incubated at 37°C for 24 h. This process was repeated five times and serially diluted (10-fold) bacterial cells were streaked on LB agar plate. Plasmid curing was confirmed by colony PCR.

To cure pFORC82_1 which harbors a gene encoding ESBL from *E. coli* FORC82 cells, we used two peptide nucleic acids (PNAs, RepE-PNA1 and RepE-PNA2), which are composed of complementary sequences including predicted ribosome binding site and start codon, respectively, against replication initiation protein E of pFORC82_1 (Panagene, Daejeon, South Korea) (Table 2). All PNAs were covalently conjugated with peptide KFFKFFKFFK to improve cell-penetrating efficiency (Good et al., 2001). *E. coli* FORC82 cells were harvested at early log phase and diluted to 10^5^ CFU/ml. The 20 μM RepE-PNA2 or the combination of PNA2 and Amp (50 μg/ml) were added to bacterial cell culture. The optical density at 600 nm of bacterial cell culture was measured every 15 min up to 7 h. The PNAs-treated cells were plated on the LB/Amp plates. Several colonies resuspended with 20 μl of PBS were spotted on LB and LB/Amp agar plate to distinguish the sensitivity against Amp. We selected clone which was sensitive to Amp and named the strain as *E. coli* PS01.

**Table 3:** Peptide nucleic acids used in this study.

PNA	Sequence (N- > C)
RepE-PNA1	KFFKFFKFFK-TCT GCT TAC CAG
RepE-PNA2	KFFKFFKFFK-CAA AGG CCT TAC

### Construction of Tn5 transposon mutant library and screening phage resistance mutants

A Tn5 transposon mutant library was generated using the EZ-Tn5 kit as described by the manufacturer (Lucigen^Ⓡ^, Middleton, WI, United States) with some modifications. Briefly, kanamycin resistance gene in plasmid pKD13 was amplified by polymerase chain reaction (PCR), wherein DNA fragment had inverted repeat sequence on both end (CTGTCTCTTATACACATCT). Purified DNA fragment (approximately 250 ng/μL) was mixed with 100% glycerol and EZ-Tn5 transposase and the mixture was incubated for 30 min at room temperature to construct transposome. One microliter of transposome was transformed by electroporation to *E. coli* PS01. To screen the phage-resistant clone, pools of transformants were mixed with EJP2 (approximately 10^9^ PFU/mL) and incubated at 30°C for 30 min and the mixture was spread on LB/Kan agar plate. Surviving colonies were isolated by streaking three times on LB/Kan agar to remove the effect of remaining phages. Phage resistance against EJP2 was confirmed by spot assay. The locus of transposon insertion was identified by whole genome sequencing (WGS) using Illumina NextSeq platform (Illumina, San Diego, CA, United States). The sequenced DNA fragments were assembled using CLC Genomics Workbench 9.0.

### Plasmid construction

The plasmids and oligonucleotides used in this study are listed in Table 1 and Table 3. Plasmid pFORC82_*waaR* (pUHE21-2 *lacI^q^*::*waaR*), which expresses *FORC82_0109* gene (putative LPS α-1,2 glycosyltransferase), was constructed using isothermal assembly with the two DNA fragments; a PCR amplified linearized pUHE21-2 *lacI^q^* plasmid, and a PCR amplified *FORC82_0109* gene. The two DNA fragments had overlapped sequences (20 bp) with each other (Gibson et al., 2009) and were inserted into the pFORC82_0109 by incubating at 50°C for 1 h in reaction buffer (25% PEG-8000, 500 nM Tris–HCl [pH 7.5], 50 mM MgCl2, 50 mM DTT, 1 mM of dNTPs, and 5 mM Nicotinamide adenine dinucleotide). The assembled plasmid was transformed to *E. coli* PS01 strain for complementation test.

### Biofilm inhibition assay

Overnight cultures of *E. coli* FORC82 and other AMR *E. coli* strains were diluted 1:100 in 2 ml of fresh LB broth. EJP2 was added at MOI 0.1 or 1 in each well of a 96-well polystyrene plate containing *E. coli* FORC82 and incubated at 25°C without shaking for 48 h. Two hundred microliter of LB broth was used as a negative control. After incubation, biofilm staining was conducted as described by Cha et al. (2019) with some modifications. All wells were washed with PBS three times to remove vegetable cells. Biofilm was fixed with 95% methanol for 15 min and stained by 0.1% crystal violet (CV) for 30 min. Each well was washed with PBS to remove the residual CV. All wells were filled with 200 μl of 33% glacial acetic acid and incubated for 45 min at RT for dissolution of biofilm. The optical density at 570 nm of each well was measured.

### Phage-antibiotic synergy

EJP2 phage was treated with cefotaxime (CTX) to investigate synergistic effect with antibiotics. Overnight-cultured *E. coli* FORC82 cells was sub-cultured 1:100 into fresh LB broth. EJP2 was treated to bacterial culture (5 × 10^6^ CFU/ml) at a MOI of 1 with or without sublethal concentration of CTX (64 μg/ml). Mixtures were incubated at 30°C for 12 h. *E. coli* FORC82 cells were obtained every 1 h up to 3 h and diluted (10-fold) bacterial cells were plated on LB plates. The number of *E. coli* FORC82 cells was determined by counting the number of colonies on LB plates. The bacterial culture treated with LB broth was used as negative control.

### Statistical analysis

GraphPad Prism 5 software was used for statistical analysis. Statistical analysis was conducted by one-way analysis of variance (ANOVA) with Tukey’s multiple comparison tests among the experimental groups (95% confidence interval). The significant differences among the experimental groups are marked with asterisks. *p* < 0.05 (*), or *p* < 0.001 (***).

## Results and discussion

### Isolation and morphological analysis of jumbo phage EJP2

**Figure 1:**
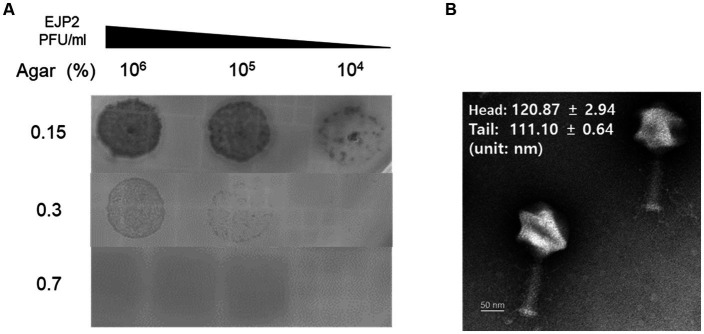
**(A)** Comparison of plaque size of EJP2 depending on the concentration of agar in LB soft agar. The concentrations of agar are shown on the left side of **(A)**. The larger plaques of EJP2 were observed at low agar concentration. **(B)** Transmission electron micrographs of phage EJP2. Phage EJP2 particles were negatively stained with 2% aqueous uranyl acetate (pH 4.0). EJP2 belongs to the *Myoviridae* family. Bar, 50 nm.

The novel *E. coli* phage EJP2 was isolated from animal feces without filtration (Supplementary Figure S1). EJP2 could infect AMR *E. coli* FORC82 strain that contains *mcr-1*-harboring plasmid (Kim J. et al., 2019) and formed small clear plaques on LB soft agar (0.7%) over the bacterial lawn of *E. coli* FORC82. EJP2 plaques were rarely visible on a high concentration of LB soft agar (0.7%) (Figure 1A), indicating that EJP2 exhibits characteristic in common with *Escherichia* jumbo phages, in which plaque size decreased as the agar concentration increased (Saad et al., 2019).

Of over 220 jumbo phages reported to date, more than over hundred phages were classified as *Myoviridae* family (Yuan and Gao, 2017). The morphological analysis using transmission electron microscopy (TEM) demonstrated that phage EJP2 belongs to the *Myoviridae* family. Both the diameter of icosahedral head (120.9 ± 2.9 nm) and the length of contractile tail (111.1 ± 0.6 nm) were over 100 nm (Figure 1B), suggesting that EJP2 has a similar morphological feature compared to large virions classified as jumbo phages.

### Bacterial growth inhibition efficacy of EJP2

**Figure 2:**
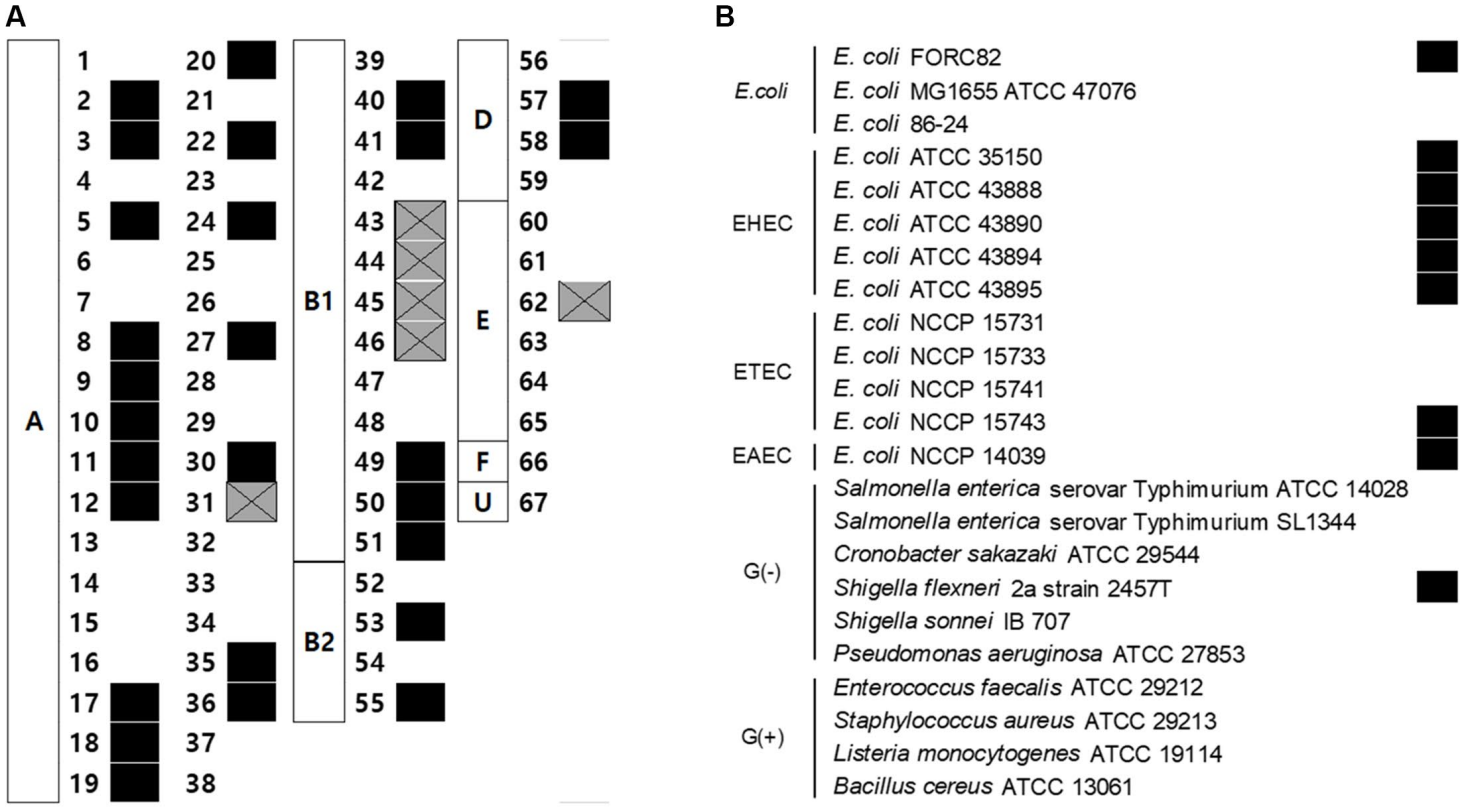
Antimicrobial spectrum of phage EJP2. **(A)** The susceptibility of 67 AMR *E. coli* against EJP2 was examined by using the spot assay. Alphabet in the white box indicates the phylogenetic groups of AMR *E. coli*. **(B)** Host range of EJP2 against pathogenic *E. coli,* other Gram-negative bacteria, and Gram-positive bacteria was determined by spot assay. Black squares indicate that EJP2 can form a plaque on the lawn of indicated bacterial strains. Gray squares with X mark present the growth inhibition zone. FORC, Food-borne pathogen Omics Research Center; ATCC, American Type Culture Collection; NCCP, National Culture Collection for Pathogens; EHEC, Enterohemorrhagic *E. coli*; ETEC, Enterotoxigenic *E. coli*; EAEC, Enteroaggregative *E. coli*; G(−), Gram-negative bacteria; G(+), Gram-positive bacteria.

Sixty-seven AMR *E. coli* isolates, pathogenic *E. coli* strains, and other Gram-negative strains were used to determine the host range of EJP2. Among 67 AMR *E. coli* strains, which were classified by the Clermont phylotyping (Clermont et al., 2013), EJP2 inhibited the growth of 33 AMR *E. coli* strains in phylogroup A (19/38), B1 (9/13), B2 (2/4), D (2/4), and E (1/6) (Figure 2A). This result indicates that EJP2 showed different host spectrum compared to those of five JEP coliphages reported by Kim et al. (2021). EJP2 showed a broader host range than those of JEP1, JEP6, JEP7 and JEP8 phages. JEP4 phage could infect AMR *E. coli* strain in phylogroup A (28/38) and D (3/4) (Kim et al., 2021), but EJP2 showed a broader inhibition spectrum against major phylogroups of AMR *E. coli* (A, B1, B2, D, and E) than JEP4 phage. EJP2 was also capable of forming plaques against Enterohemorrhagic *E. coli* (EHEC), Enterotoxigenic *E. coli* (ETEC), Enteroaggregative *E. coli* (EAEC), and *Shigella flexneri* (Figure 2B). In bacterial challenge assay, EJP2 could retard the growth of *E. coli* FORC82 4 h after infection at 30°C and 2 h after infection at 37°C (Supplementary Figure S2). The broad host spectrum of EJP2 implies its potential utility as biocontrol agents in clinical or food applications.

### Genomic and phylogenetic analysis of EJP2

**Figure 3:**
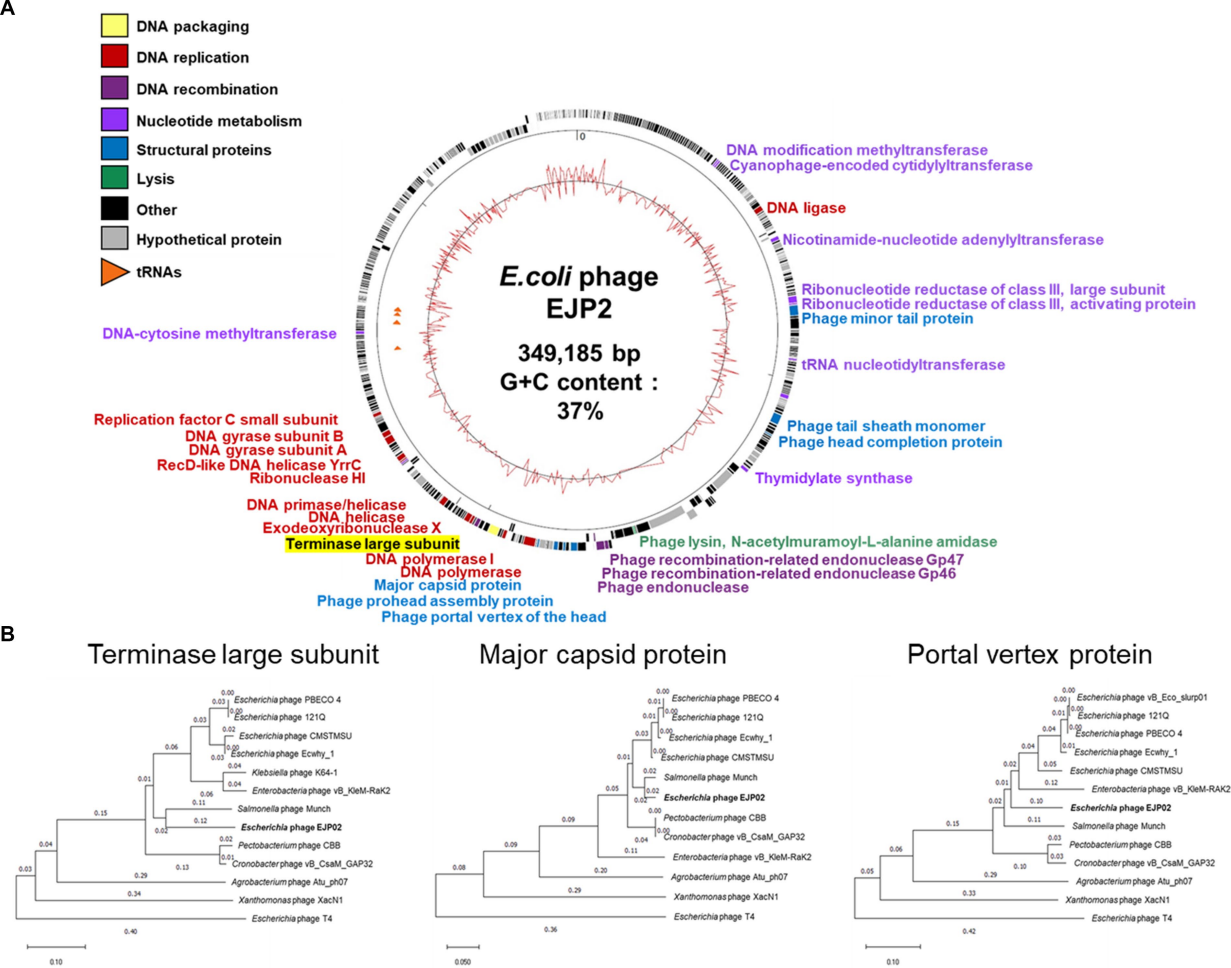
**(A)** Whole-genome map of phage EJP2. Predicted ORFs with the corresponding gene products are arranged on the EJP2 genome. Functional groups are categorized into colors. The GC content is presented as the inner track. **(B)** Phylogenetic tree comparing the terminase large subunit, major capsid protein, and portal vertex protein of phage EJP2 among other jumbo phages. Jumbo phages were selected as they share homology with the amino acid sequences of indicated protein using BlastP. Phage T4 was selected as an outgroup phage that is not closely related to the corresponding sequence of EJP2. Sequence relationships were inferred using the neighbor-joining method and evolutionary distances were computed using *p*-distance method by MegaX software.

Genomic characteristics of phage EJP2 were identified through whole-genome analysis. EJP2 possesses a 349,185 bp circular double-stranded DNA genome with average G + C content 37% and was thus classified as jumbo phage. EJP2 contains 540 putative ORFs and 6 genes encoding tRNAs (Figure 3A). Most of predicted genes (471 ORFs) encode hypothetical proteins with unknown functions. Of the 540 putative ORFs, only a small subset (12.8%, 69/540) were assigned putative functions (Supplementary Table S1). The analysis of phage life cycle is essential for developing phage therapeutic agent because temperate phages have the inherent capacity to transfer genes associated with bacterial virulence or antibiotic resistance by transduction (Howard-Varona et al., 2017). No genes associated with lysogenization, such as integrase, excisionase, transposase, superinfection immunity, repressor, and genome attachment site (attP) were predicted in the EJP2 genome, suggesting that EJP2 may be a virulent phage (Fogg et al., 2011; Davies et al., 2016). In addition, antibiotics resistance and virulence-associated genes were not identified in EJP2. BlastN analysis revealed that the whole genome of phage EJP2 shares less than 14% nucleotide identity with registered *E. coli* jumbo phages. Dot plot analysis presented low sequence homology with five *Escherichia* jumbo phages and one *Salmonella* jumbo phage at both the nucleotide and amino acid level (Supplementary Figure S3).

As different from the small phage genomes, the putative functional genes of EJP2 were scattered throughout its genome. The function of ORFs were categorized into 6 groups; nucleotide metabolism, DNA replication, DNA recombination, DNA packaging, structure proteins, and lysis (Figure 3A). One noticeable features of jumbo phages is that they possess their own enzymes for DNA replication, recombination, and transcription, capable of self-replication independent of host machinery (Iyer et al., 2021). In EJP2 genome, four ORFs (EJP02_150, EJP02_151, EJP02_301, and EJP02_303) were annotated as ribonucleotide reductase subunit whose composes enzyme for synthesis of deoxyribonucleotides from ribonucleotides with the help of glutaredoxin 1 (EJP02_192) and thioredoxin (EJP02_485) (Dwivedi et al., 2013; Sengupta and Holmgren, 2014). Two ORFs were predicted as DNA polymerase (EJP02_261) and DNA polymerase I (EJP02_270) (Supplementary Table S1). EJP2 also encoded other 9 genes associated with DNA replication and 3 genes associated with DNA recombination, respectively. In addition to various genes involved in DNA metabolism, EJP2 possesses two proteins (RNA polymerase sigma factor D and RNA ligase) for its own transcription (Supplementary Table S1). Overall, the results of genome annotation of EJP2 suggest the evolution of EJP2 toward reduced dependency on the host bacterium. This finding would be helpful to explain the broad host spectrum of phage EJP2.

The MCP, TerL protein and portal vertex protein are major conserved proteins in phage genomes and are thus used as phylogenetic markers to organize the phage families through single gene analysis (Smith et al., 2013; Prevelige and Cortines, 2018). Analysis of phylogenetic trees based on these three genes revealed that EJP2 is grouped with Rak2-like phage family (Šimoliūnas et al., 2013), which includes *E. coli* phage PBECO4 (Kim et al., 2013), *E. coli* phage 121Q (accession number: NC_025447), *Cronobacter* phage vB_CsaM_GAP32 (Abbasifar et al., 2014), *Enterobacteria* phage vB_PcaM_CBB (Buttimer et al., 2017), and *Salmonella* phage Munch (accession number: MK268344.1). EJP2 appeared to be evolutionally closest to *Salmonella* phage Munch for three proteins (Figure 3B).

### Phage EJP2 receptor analysis using Tn5 insertion mutant library

**Figure 4:**
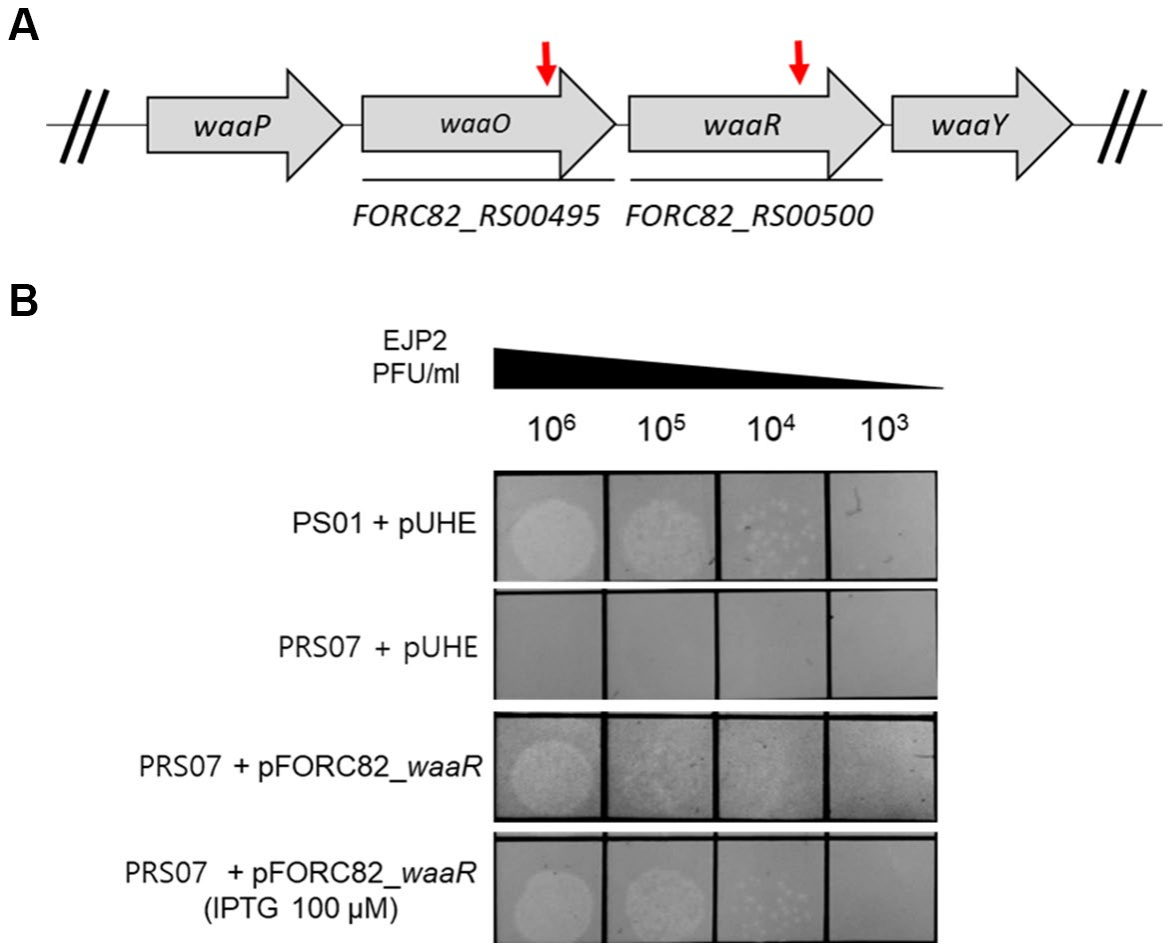
EJP2 receptor analysis. Schematic representation of the genes associated with LPS biosynthesis in *E. coli* FORC82 **(A)**. Red arrows indicate genes disrupted by transposon insertion. Locus tags of genes were presented below each arrow. Complementation of the LPS biosynthesis gene (putative *waaR*, *FORC82_RS00500*) to identify the restored susceptibility against EJP2 in *E. coli* PS01 **(B)**. The concentration of IPTG is presented in parentheses. LPS is a phage EJP2 receptor. One representative result of triplicate experiments is shown.

The host of EJP2, *E. coli* FORC82, harbors three F+ plasmids encoding various antibiotics resistance genes (Kim J. et al., 2019). We conducted plasmid curing by using acridine orange and PNA treatments to remove pFORC82_1, which carries most AMR genes. Unexpectedly, acridine orange treatment cured the pFORC82_2 (data not shown). Treatments of PNAs targeting RBS or start codon of plasmid replication protein (RepE) resulted in *E. coli* PS01 that was sensitive to β-lactam antibiotics (Supplementary Figure S4A). *E. coli* PS01 showed no difference in phage sensitivity compared to the *E. coli* FORC82 (Supplementary Figure S4B). We confirmed the deletion of about 6.3 kb region containing class A extended-spectrum β-lactamase (*bla*_CTX-M-65_) gene between IS26 family transposase genes (Tnp26), which is known to form transposons carrying antibiotic resistance genes (Harmer and Hall, 2016; Supplementary Figure S4C). A Tn5 insertional mutant library of *E. coli* PS01 was constructed to screen for phage EJP2 resistance. Two EJP2-resistant colonies were obtained and Tn5 insertion sites were identified by WGS as putative *waaO* (*FORC82_RS00495*, LPS 3-α galactosyltransferase) and putative *waaR* (*FORC82_RS00500,* LPS α-1,2 glucosyltransferase), both of which are associated with biosynthesis of LPS (Figure 4A). LPS 3-α galactosyltransferase is an enzyme that adds the galactose (GalI) to the first glucose (GlcI) to form branches of LPS (Pradel et al., 1992). Other reports describe that *waaO* gene encodes LPS α-1,3-glucosyltransferase or LPS α-1,3-galactosyltransferase, which adds hexose II residue to glucose I of outer core of LPS (Shibayama et al., 1998; Qian et al., 2014). LPS α-1,2 glucosyltransferase is an enzyme that adds the third glucose (GlcIII) to the second glucose (GlcII) in the outer core of *E. coli* LPS (Shibayama et al., 1999). Phage resistant mutant with Tn5 insertion in putative *waaR* gene was designated as *E. coli* PRS07. The susceptibility of *E. coli* PS01 and *E. coli* PRS07 to EJP2 was examined by spot assay. As expected, EJP2 could not form plaques on the lawn of *E. coli* PRS07 (Figure 4B). When the putative *waaR* gene was complemented using inducible plasmid pUHE21-2 *lacI*^q^, the sensitivity of EJP2 against PRS07 was completely restored, even without IPTG induction (Figure 4B). These results indicated that EJP2 recognizes LPS as a phage receptor and a third glucose of LPS outer core, or O-antigen would be required for EJP2 infection. LPS produced by *E. coli* varies depending on the pathogen type and this is due to the diversity in O-antigen structure (Liu et al., 2020). Moreover, the core region of LPS is known to have five different types and distribution of LPS core types is diverse among different phylogroups (Leclercq et al., 2021). Because of these differences in LPS structure, the sensitivity against EJP2 was different among *E. coli* strains (Figure 2).

### Biofilm inhibition assay

**Figure 5:**
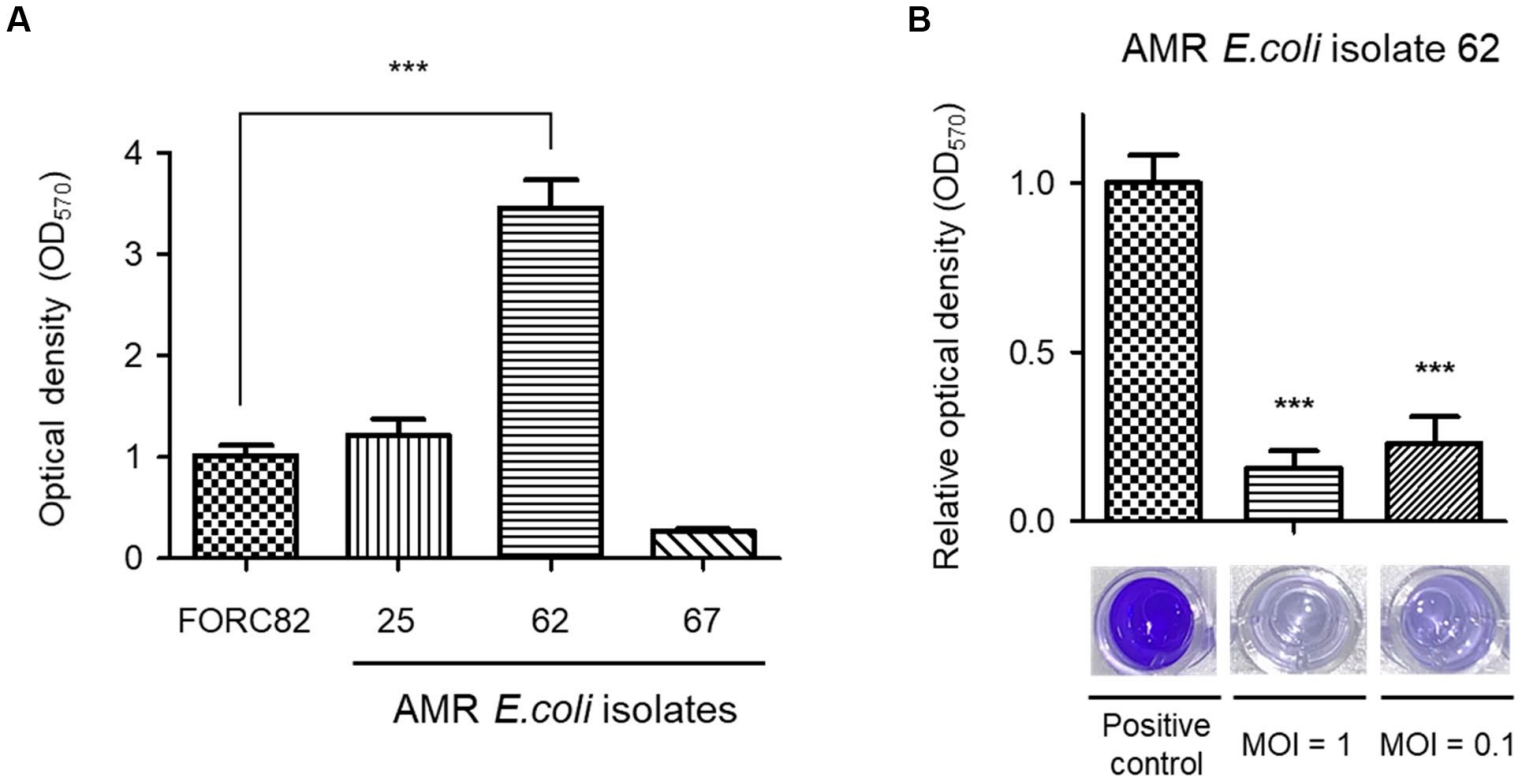
Comparison of biofilm formation of 4 AMR *E. coli* strains in 96-well polystyrene microplates **(A)**. Biofilm removal efficacy of phage EJP2 **(B)**. Biofilms of AMR *E. coli* isolate 62 were treated with EJP2 at a MOI of 1 and 0.1 and incubated for 48 h. Each column represents the mean of triplicate experiments, and error bars indicate the standard deviation. One representative result of triplicate experiments is shown. ***p < 0.001.

Biofilm formation by AMR *E. coli* cells can pose a serious threat in human health and food industry because this structure is generally resistant to the human immune system and shows increased-tolerance against antibiotic treatments (Sharma et al., 2016; Zhou et al., 2022). Bacteriophages are known to efficiently remove biofilms (Pires et al., 2017), and the ability of EJP2 to eradicate biofilms formed by AMR *E. coli* was tested. Before phage treatment, we tried to screen AMR *E. coli* strain whose biofilm-forming ability was higher than *E. coli* FORC82. AMR *E. coli* isolate 62 showed significantly higher biofilm formation on polystyrene surfaces than *E. coli* FORC82 (Figure 5A), thus we used *E. coli* isolate 62 for biofilm inhibition assay. EJP2 was added to each well at MOI 1 and 0.1 and its treatment reduced biofilm formation by more than 80% compared to the positive control (Figure 5B). These results imply that anti-biofilm capacity of EJP2 would be helpful in reducing disease caused by AMR *E. coli* infection such as catheter-associated urinary tract infection.

### EJP2 and CTX synergy against AMR *Escherichia coli* FORC82

**Figure 6:**
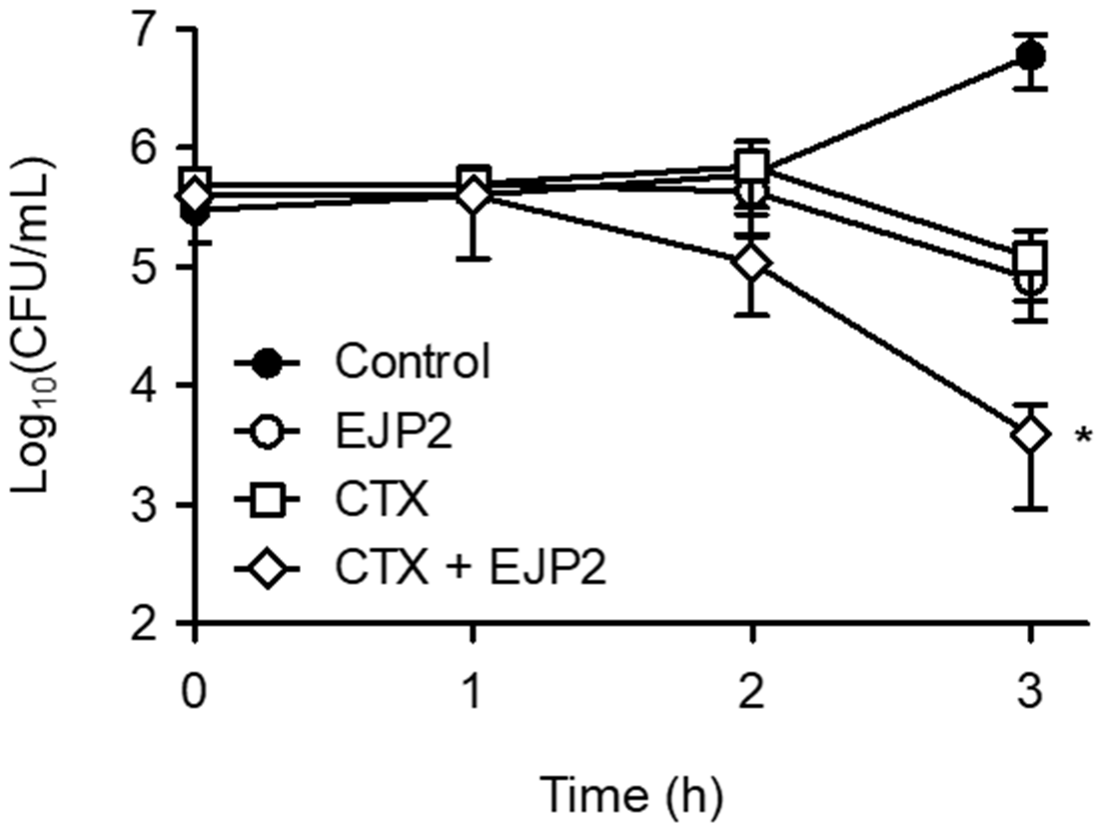
EJP2 and CTX synergy against *E. coli* FORC82. The antimicrobial activity of single treatment (EJP2, or CTX), and their combination were evaluated. Each Colony Forming Unit (CFU) was numerated after 3 h inoculation. The means with standard deviation of triplicate experiments are shown. *p < 0.05.

CTX, one of third generation cephalosporins, was selected to investigate its synergistic antimicrobial effects. CTX is considered by WHO as the “highest priority critically important antimicrobials” for human medicine (World Health Organization, 2017) and the *E. coli* FORC82 was highly resistant to CTX (MIC ≥128 μg/ml, data not shown). The combination of EJP2 and sublethal concentration of CTX (64 μg/ml) significantly reduced *E. coli* FORC82 population after 3 h of treatment compared to the separate treatment of EJP2 or CTX (Figure 6). Two studies reported synergistic bacterial lysis by combining CTX treatment with T4 and two other T4-like phages, CTX treatment shortened the latent period of those phages (Comeau et al., 2007; Ryan et al., 2012). The present study revealed that EJP2 recognizes LPS as a phage receptor (Figure 4). LPS is considered to be a permeability barrier against hydrophilic and hydrophobic compounds (Lehman and Grabowicz, 2019) and it is well-known that LPS truncation or modification increases susceptibility of *E. coli* against antibiotics, and detergents. LPS truncation caused by T4 phage infection led to hypersensitivity against food grade surfactant (Zhong et al., 2020) and rough type LPS by r*fa* gene knockouts increased sensitivity of *E. coli* to colistin (Burmeister et al., 2020). Similar to these findings, the increased susceptibility of *E. coli* FORC82 to CTX in combination treatment with EJP2 may be due to LPS modification caused by EJP2 infection, as bacteria generally modify their receptors to avoid the phage infection (Labrie et al., 2010). The synthesis of at least the third glucose in outer core of LPS is necessary for EJP2 infection (Figure 4), it is possible that EJP2-resistant *E. coli* FORC82 may have rough type LPS, potentially making CTX more accessible to outer membrane proteins, such as OmpC, and OmpF (Goltermann et al., 2022; Masi et al., 2022). The observed synergy between EJP2 and CTX suggests that EJP2 could be used as alternative and/or adjuvants to antibiotics, potentially reducing the use of antibiotics. For better understanding the mechanism of action about synergistic antimicrobial effects, further investigation is needed.

## Conclusion

In this work, we have described AMR *E. coli*-specific jumbo phage EJP2 which was isolated from animal feces. EJP2 belongs to the *Myoviridae* family with a head and tail size of over 100 nm. Its genome contains 349,185 bp with 540 ORFs encoding genes for DNA replication, DNA recombination and nucleotide metabolism, DNA packaging, structural proteins, lysis, 6 tRNAs, and many genes whose functions remain unknown. Phylogenetic analyses of TerL, MCP, and portal vertex protein places EJP2 in a clade similar to *E. coli* and *Salmonella* jumbo phages, but with low sequence homology, suggesting a novel lineage for EJP2. Phage EJP2 exhibits a wide host range, biofilm removal activity, and synergistic effect with CTX against AMR *E. coli*, making it potentially a good candidate for the development of a biocontrol agent against diseases caused by AMR *E. coli* strains.

## Data availability statement

The datasets presented in this study can be found in online repositories. The name of the repository and accession number can be found below: NCBI: OQ411014.

## Author contributions

DJ, HK, and SR conceived and designed the experiments. DJ and HK carried out the main body of research, performed the experiments and bioinformatics analysis, and wrote the manuscript. DJ and YL contributed in phage isolation. JK contributed in provide a source and an information about AMR *E. coli* FORC82. SR supervised the work progress and edited the manuscript. All authors have read and approved the final manuscript.

## Funding

This research was supported by a grant of the Korea Health Technology R&D Project through the Korea Health Industry Development Institute (KHIDI), funded by the Ministry of Health & Welfare, Republic of Korea (grant number: HI21C0901).

## Conflict of interest

The authors declare that the research was conducted in the absence of any commercial or financial relationships that could be construed as a potential conflict of interest.

## Publisher’s note

All claims expressed in this article are solely those of the authors and do not necessarily represent those of their affiliated organizations, or those of the publisher, the editors and the reviewers. Any product that may be evaluated in this article, or claim that may be made by its manufacturer, is not guaranteed or endorsed by the publisher.

## Footnotes

^1^https://rast.nmpdr.org/
