# Supplementary material for: Characterization and genomic study of EJP2, a novel jumbo phage targeting antimicrobial resistant Escherichia coli
Source: Front Microbiol. 2023 May 12;14:1194435. doi: 10.3389/fmicb.2023.1194435 (PMC10213699; doi:10.3389/fmicb.2023.1194435)
Supplement: Supplementary file 1 [file Data_Sheet_1.PDF]

Characterization and Genomic study of EJP2, a Novel Jumbo Phage Targeting Antimicrobial Resistant *Escherichia coli*

Dohyeong Jo†, Hyeongsoon Kim†, Yoona Lee, Jinshil Kim and Sangryeol Ryu*

Department of Food and Animal Biotechnology, Department of Agricultural Biotechnology, Research Institute of Agriculture and Life Sciences, Seoul National University, Seoul 08826, Korea

* Corresponding author.

Mailing address: Department of Food and Animal Biotechnology, Seoul National University, Seoul 08826, Korea

Phone: 82-2-880-4856. Fax: 82-2-873-5095. E-mail: sangryu@snu.ac.kr

†These authors have contributed equally to this work

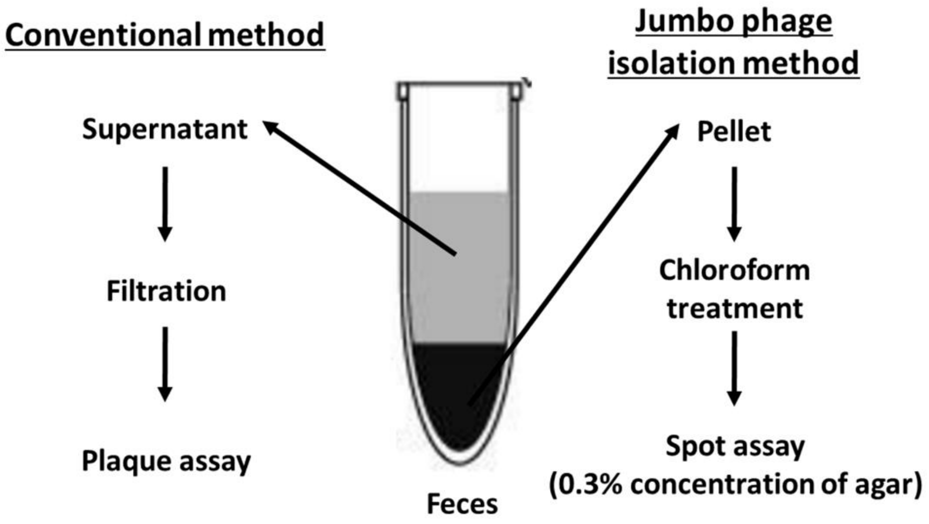

Figure S1. Schematic of jumbo phage isolation. As different from conventional methods that use supernatants from samples with filtration, large particles of feces were separated by centrifugation and suspended with SM buffer. The resultant lysate was used for spot assay using low concentration of agar (0.3%). Small plaques were picked and used for further purification.

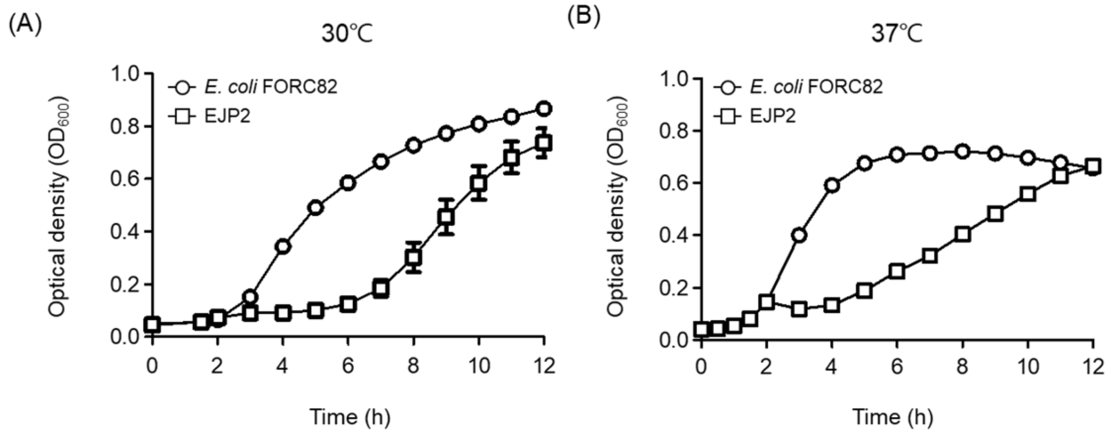

Figure S2. Bacterial growth inhibition assay against *E. coli* FORC82. Bacterial cells were prepared at 30℃ (A), and 37℃ (B). EJP2 was inoculated into bacterial culture at a MOI of 1. The optical density at 600 nm was measured every 1 h for 12 h. The control group without phage (○) and the experimental group with phage EJP2 (□). The data shown are the mean values from three independent experiments, and the error bars represent the standard deviations.

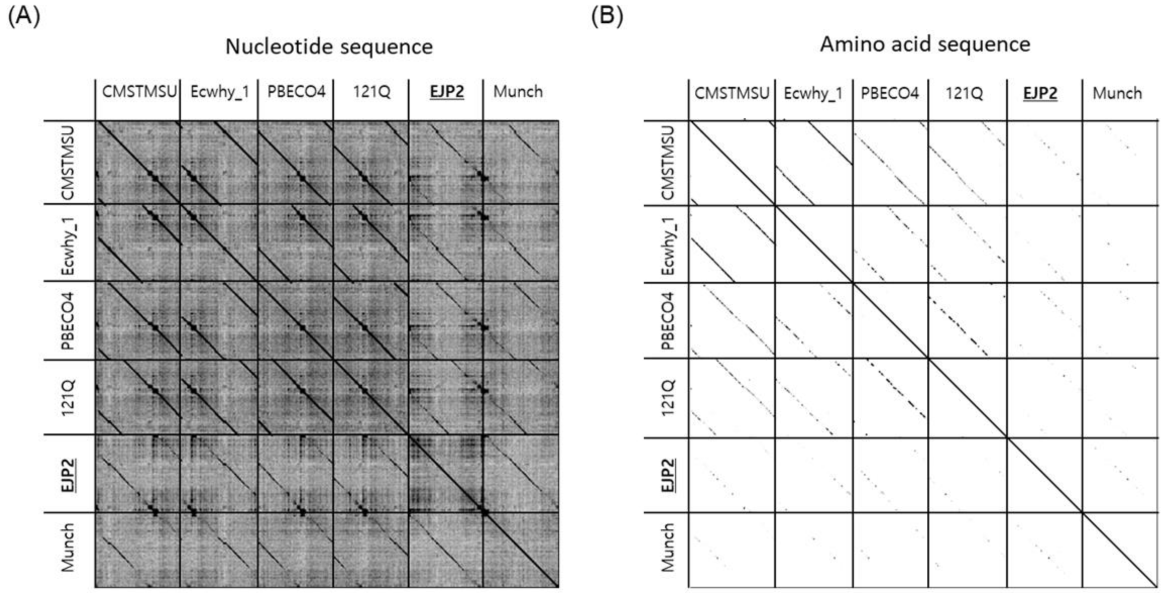

Figure S3. Comparative genomic analysis of EJP2. The overall genomic and proteomic similarity among EJP2 and other six jumbo phages were identified by dot plot analysis. Dot plots were generated using Gepard v1.40 based on the nucleotide sequences (A) and the amino acid sequences (B) of EJP2 and other six jumbo phages. Black lines represent similarities among phage nucleotide or amino acid sequences.

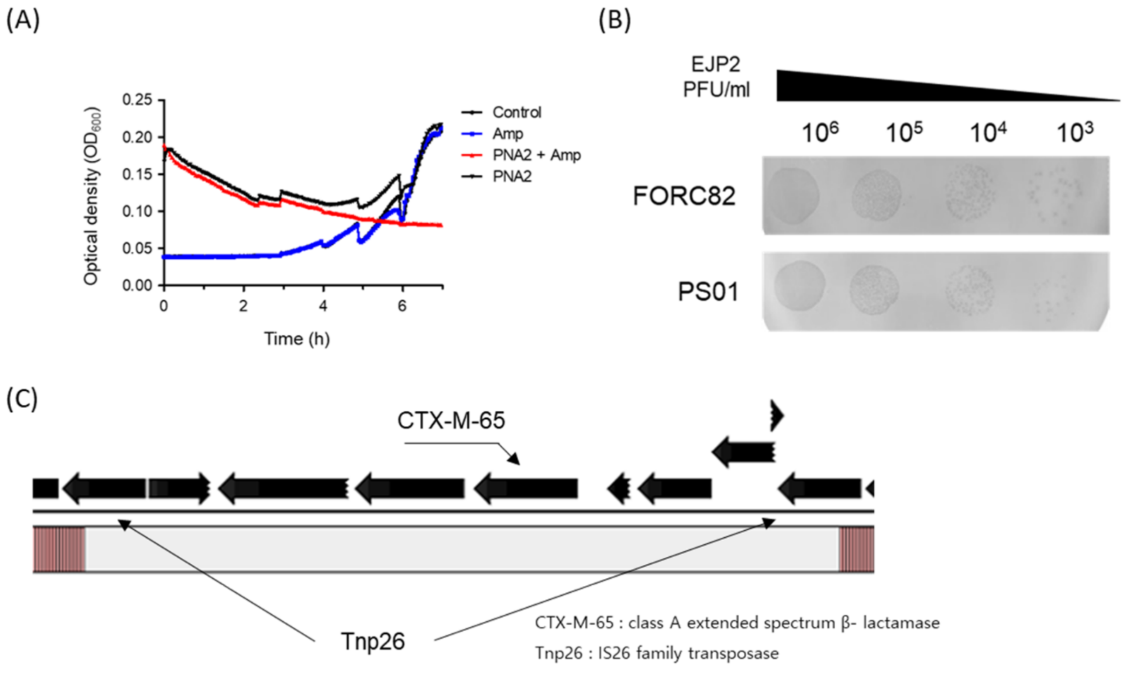

Figure S4. PNA treatment for curing pFORC82_1. (A) Restoration of sensitivity to AMP by PNA2 treatment. (B) Deletion of about 6.3 kb region containing class A extended-spectrum β-lactamase (blaCTX-M-65) gene in pFORF82_1. Red color under the thick black arrows indicates matching sequence with reference sequence, and grey color represents deleted sequence. CTX-M-65, class A extended-spectrum beta-lactamase; Tnp26, IS26 family transposase. (C) Same efficiency of plating (EOP) of *E. coli* FORC82 and *E. coli* PS01 against EJP2. The susceptibility of two strains against EJP2 was measured by spot assay.
